# Legal Limits Relaxed: Time to Look at Other Barriers Faced by Women Seeking Termination of Pregnancy for Fetal Anomalies

**DOI:** 10.7759/cureus.34144

**Published:** 2023-01-24

**Authors:** Rimpi Singla, Nasreen T Banu, Aashima Arora, Neelam Aggarwal, Madhu Gupta

**Affiliations:** 1 Obstetrics and Gynaecology, Postgraduate Institute of Medical Education and Research, Chandigarh, IND; 2 Community Medicine & School of Public Health, Postgraduate Institute of Medical Education and Research, Chandigarh, IND

**Keywords:** abortion law, delay to diagnosis, fetal anomalies, barriers to abortion, elective abortion

## Abstract

Introduction

Advancements in prenatal diagnostic techniques have led to an increase in demand for termination of pregnancy for fetal anomalies (TOPFA). While relaxation in the legal gestational age limits across various countries relieves an important barrier, there is a need to identify the reasons that lead to delays in seeking abortion for fetal anomalies, because abortion-related complications increase with gestational age.

Methods

In this hospital-based qualitative study, antenatal women referred to a tertiary care institute in North India because of major fetal anomalies were explained about the study. Those women who fulfilled the inclusion criteria were recruited after taking consent. Details of antenatal care and prenatal tests were recorded. An in-depth inquiry was made into the reasons for the delay in prenatal tests, the delay in the decision for abortion, and specific problems that they faced in seeking TOPFA.

Results

Out of 80 women who met the inclusion criteria and consented to participate, more than 75% had received antenatal care in public healthcare facilities. Less than 50% of women received folic acid in the first trimester while 26% had first contact with healthcare facilities in the second trimester. Only 21 women underwent screening for common aneuploidies. Second-trimester anomaly scan was delayed in 35 women due to women-centered reasons (n = 17) or provider-centered (n = 19) reasons. Only 37.5% of women were counseled about fetal anomalies by their primary care provider. Owing to delay at multiple levels, 40 women (50%) could receive counseling about fetal abnormality for the first time after 20 weeks. These women could not be offered abortion because this study was carried out before the amendments in the Medical Termination of Pregnancy Act in India. The older act allowed abortion up to 20 weeks of gestation. Seventeen women could obtain permission for an abortion from a court of law. Arrangements for travel and stay and dependence on family members were the main problems faced by women seeking TOPFA.

Conclusions

Delay in diagnosis of a fetal anomaly due to delay in seeking antenatal care, irregular follow-up, and lack of pre-test counseling are the major reasons for the delay in the decision for abortion. This is further compounded by inadequate post-test counseling. Lack of awareness, failure or delay in counseling, need to travel to another facility for abortion, dependence on family members, and financial issues are the major barriers.

## Introduction

With improvement in the management of prematurity, asphyxia, and sepsis, birth defects are emerging as a significant cause of neonatal mortality in developing countries. According to one study, the mortality rate was 30.1% among 976 children with birth defects compared to 0.25% among 61,568 children born free of birth defects [1]. When such a child survives with a major disability, the family has to endure mental trauma and financial burden also.

Improvements in antenatal testing have enabled early diagnosis of chromosomal and structural abnormalities, but in-utero treatments are yet not freely available in developing countries. Moreover, most of the anomalies are not amenable to in-utero treatment. The option of termination of pregnancy for fetal anomalies (TOPFA) should be an integral component of counseling while offering prenatal testing [2], but abortion laws vary across the globe. Only 115 (57%) countries have legal provisions for abortion for fetal abnormalities [3]. In India, the Medical Termination of Pregnancy (MTP) Act, which came into force in 1971, allowed abortion for various indications (including fetal anomalies) till 20 weeks [4]. An increase in demand for TOPFA due to advancements in prenatal diagnostic techniques has forced the liberalization of abortion laws in some countries. In India, too, the upper limit of gestational age for abortion for fetal anomaly has been relaxed via amendments in MTP Act in 2021 [5].

Despite liberal law, out of an estimated 15.6 million abortions in India in 2015, 11.5 million (73%) abortions were done outside health facilities, and 0.8 million (5%) were carried out by unsafe methods [6]. Thus, the gestational age limit imposed by law is not the only barrier to safe abortion. Access to abortion, too, needs to be improved [7]. Fetal malformations are mostly detected in the second trimester, which makes access to abortion even more difficult because the already narrow provider base shrinks further when it comes to second-trimester abortions [8]. Relaxation in legal gestational age limits should not be viewed as liberty to delay abortion because abortion-related complications and psychological challenges the patient faces are directly proportional to gestational age.

This study was conducted in a tertiary care institute in Chandigarh, a North Indian union territory. The majority of abortions are sought here for fetal anomalies. Women reach here from far-off places after being refused abortions at nearby hospitals. Many women come after 20 or even 24 weeks of gestation for malformations that could have been detected much earlier. Sometimes women are not even aware of the reason for which they have been referred. Hence, this study was initiated as a response to the problems faced by women in accessing abortion for fetal anomalies. The aim of this prospective study was to have insight into the reasons that lead to delays in the diagnosis of abnormalities, problems faced by women in accessing prenatal tests and deciding on termination of pregnancy, and the problems they encountered for having an abortion for this unambiguous indication.

## Materials and methods

Ethical considerations

Approval from the Institutional Ethics Committee, Postgraduate Institute of Medical Education and Research, Chandigarh (INT/IEC/2019/001942) was obtained before initiating the study. Written informed consent was obtained from all the participants.

Study design, health facility, and study population

This prospective study was conducted in the outpatient department (OPD) of the department of obstetrics and gynecology in a tertiary care institute in Chandigarh, a North Indian union territory. All antenatal women attending the antenatal clinic or medical termination of pregnancy (MTP) clinic because of major fetal anomalies were explained about the study and invited to participate from July 2019 to December 2020. Women approaching MTP for other indications, women enrolled in other research projects, and women not willing to provide consent were excluded. Every year approximately 150-160 women approach the institute for MTP for fetal anomalies. However, due to the coronavirus disease 2019 (COVID-19) pandemic and restricted OPDs, this number reduced drastically during this period. All the eligible women who reported in the clinics were included. A purposive sampling technique was used.

Data collection methods

Baseline demographic details, history, and examination findings were recorded on a pre-designed pretested interview schedule with closed and open-ended questions. Socioeconomic status (SES) was determined by the modified Kuppuswamy SES scale 2019 [9]. Women were asked about details of antenatal care that they received in their current pregnancy, the place of antenatal care (public or private facility), gestational age when pregnancy was confirmed, duration between confirmation of pregnancy and seeking antenatal care, history of intake of folic acid, exposure to teratogens, and fetal outcome in previous pregnancies using a structured form.

Open-ended questions were asked regarding reasons for the delay in seeking antenatal care, details of investigations pertaining to screening and diagnostic tests, date of advice for the tests, any delay and specific difficulties faced in screening/diagnostic tests, reason(s) for delay (if so), whether counseled by the physician about the fetal diagnosis, and whether there was any delay in counseling and reasons for the same. To supplement this information, prescription slips and test reports were carefully checked for dates of antenatal visits, the content of advice, and the dates on which the tests were done. Delay in seeking TOPFA was defined as seeking termination after 20 weeks [4].

Ethically, after the data collection, requisite antenatal investigations were carried out, and abortion was provided/or denied according to the provisions of the MTP Act [4].

Analysis

The normalcy of continuous variables was checked by using the Kolmogorov-Smirnov test. Pearson's chi-squared test was used to determine whether there was a statistically significant relationship between categorical variables, such as age and SES, with the delays in prenatal testing. Fisher's exact test was used to explore the association between the delay at various levels with baseline parameters, wherever more than 20% of the total number of cells had an expected count of less than five. The p-value < 0.05 was considered significant. Manual thematic analysis was done for qualitative textual data.

For the textual data, the reason(s) for delay were stratified into three categories: patient-centered, provider-centered, or administration (system)-centered factors. A further reason for referral to our center, especially the reason cited by the provider for not doing MTP, was enquired about and checked from the referral slip, if available. All the women were asked whether and what problems they faced in deciding on abortion and accessing abortion services. Answers to all the questions were left open-ended.

## Results

Study population

We invited 100 consecutive women to participate in the study. Out of these, 20 women were excluded because abortion was sought for indications other than fetal anomalies (n = 8) or they had enrolled in some other research project (n = 6), or were unable to provide information because of psychological stress (n = 4); two women were excluded because questionnaire could not be completed (n = 2). Data from 80 women were available for analysis.

Demographic data and history

The mean age of the study population was 26.89 ± 4.22 years. Most women belonged to the middle socioeconomic class, and >70% had received education till high school or senior school. Obstetric history has been detailed in Table 1. Women with medical disorders had been well-controlled or in remission at the time of conception and during pregnancy. None of them had been taking any teratogenic drug. Routine antenatal investigations were normal (Table 1).

**Table 1:** Baseline data: demography, obstetric history, examination, and investigations BP: blood pressure; SD: standard deviation; IQR: interquartile range (1st and 3rd quartile); TLC: total leukocyte count; TSH: thyroid-stimulating hormone.

Parameter	Value
Demographic data	
Socioeconomic status, n (%)	
Upper lower	13 (16.3)
Lower middle	29 (36.3)
Upper middle	32 (40.4)
Upper class	6 (7.5)
Education, n (%)	
Illiterate	2 (2.5)
Primary	1 (1.3)
Middle	5 (6.3)
High school	29 (36.3)
Senior school	30 (37.5)
Graduate	13 (16.3)
Residence, n (%)	
Urban	29 (36.3)
Rural	51 (63.8)
Obstetric history, n (%)	
Primigravida	43 (53.8)
Multigravida	37 (46.3)
History of abortions	17 (21.3)
Intrauterine fetal demise or infantile death	7 (8.8)
Fetal structural or chromosomal abnormalities	3 (3.8)
Past history, n (%)	
Pre-existing medical disorders	14 (17.5)
Examination	
Pulse rate (median, IQR), bpm	82 (78, 86)
Systolic BP (median, IQR), mmHg	100 (100, 110)
Diastolic BP (median, IQR), mmHg	76 (70, 80)
Investigations	
Hemoglobin (mean ± SD), gm/dl	10.2 ± 1.2
TLC (mean ± SD), cells per cubic mm	9031 ± 1514
Platelet count (mean ± SD), lakh/cubic mm	2.0 ± 0.63
Fasting blood sugar (mean ± SD), mg%	85.8 ± 9.8
TSH (mean ± SD), mIU/L	1.9 ± 1.3

Details of fetal surveillance

Most women had been receiving antenatal care in public healthcare facilities (Table 2). More than 50% of women were deprived of folic acid supplementation in the first trimester.

**Table 2:** Details of antenatal care received IQR: interquartile range (1st and 3rd quartile).

Parameter of antenatal care	Value
Antenatal care from a public facility, n (%)	62 (77.5)
Gestation at confirmation of pregnancy, mean ± SD (weeks)	7.44 ± 2.976
The time between confirmation of pregnancy and seeking care, median (IQR) (days)	11 (2,29)
Folic acid prescribed, n (%)	51 (63.8)
Folic acid received, n (%)	39 (48.8)
Pre-conceptional folic acid received, n (%)	4 (5.0)

Only 21 women underwent screening tests for aneuploidies, out of which three had a high risk of abnormalities. Two women had an increased risk of neural tube defects for which targeted ultrasound was done, and one had an increased risk of trisomy 21 for which amniotic fluid analysis was done.

**Figure 1:**
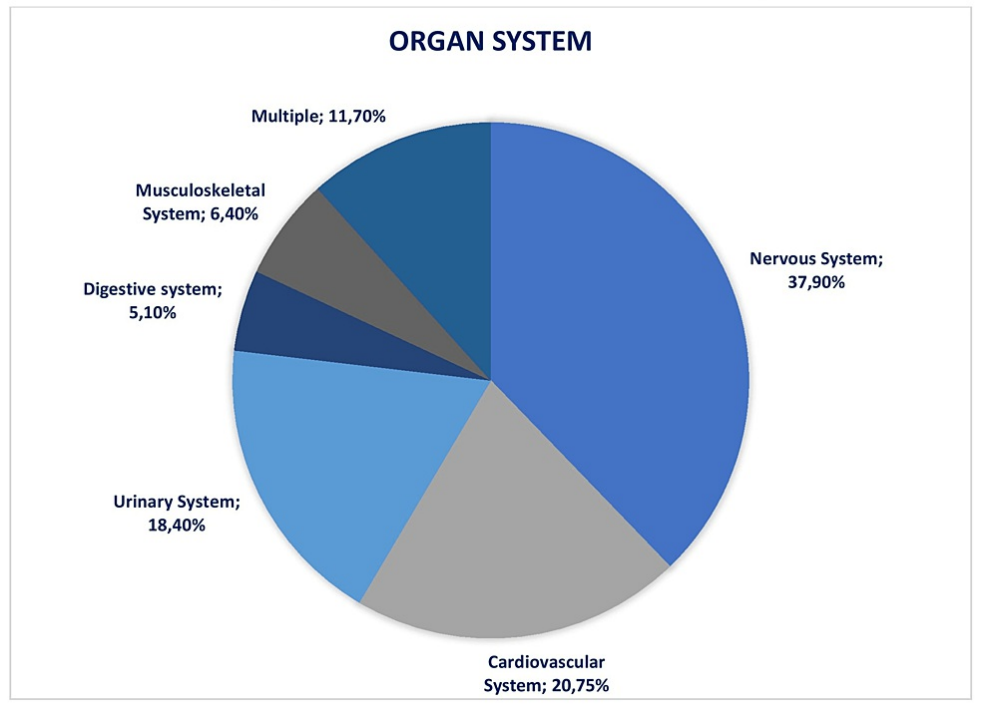
Distribution of fetal anomalies according to the organ system involved Values are given as numbers and percentages.

The most common organ system in fetuses to have malformation was the nervous system (37.90%), followed by the cardiovascular system (20.75%) (Figure 1).

Details of delay in the diagnosis of fetal anomalies

The delay in diagnosis started with the delay in registering for antenatal care. More than 25% of women had first contact with a healthcare facility after the end of the first trimester, while four women entered the antenatal care program after 20 weeks. Screening for common aneuploidies was missed in >73% of women (Table 3) mainly because of women-centered reasons. Diagnostic prenatal tests (second-trimester ultrasound and amniocentesis) were delayed in 35 women (Table 3). It was done after 20 completed weeks in 31 cases (39%). In four cases, the ultrasound was done in time, but the physician advised to repeat the ultrasound after one month. In these women, there was no change in sonographic findings. The reasons for the delay were women-centered in 17 cases and provider-centered in 19 cases. Among patients who did not get the ultrasound done timely despite being advised by the physician, four could not find time from domestic engagements, and one had no family member free to accompany them. When asked about the reason for the delay in the ultrasound, a few women said, "we need to travel to a nearby city for an ultrasound, and who would do household work in my absence." Few patients had delays due to a combination of multiple factors (personal, provider-related, and logistic barriers). One woman said, "Doctor did not say that delay would lead to so many problems…. Further COVID lockdown was imposed."

**Table 3:** Reason for delay in seeking abortion * Diagnostic prenatal test includes second-trimester scan and amniocentesis. IQR: interquartile range (1st and 3rd quartile).

Level of delay	
Aneuploidy screening not done, n (%)	59 (73.7)
Women-centered reasons, n (%)	32 (54.2)
Did not report at appropriate gestation, (n)	15
Did not get the screening test done, (n)	17
Provider-centered reasons, n (%)	22 (37.3)
Not advised by the physician, (n)	19
Lack of pre-test counseling, (n)	3
Administration/system-centered reasons, n (%)	5 (8.47)
Lockdown (n)	5
Multiple reasons (not available locally and financial problems and lockdown), n (%)	5 (8.47)
Delay in diagnostic prenatal test^*^	
Gestation at second-trimester scan, mean ± SD (weeks)	19.5 ± 2.02
Reason for delay in prenatal diagnostic test (n = 35)	
Women-centered reasons, n (%)	17 (48.57)
Did not report at appropriate gestation, (n)	12
Not done despite advice, (n)	5
Provider-centered reasons, n (%)	19 (54.3)
Lack of pre-test counseling, (n)	12
Delay in advice from the physician, (n)	7
Administration/system-centered reasons, n (%)	6 (17.1)
Non-availability of ultrasound facility locally, (n)	2
Travel restrictions imposed by lockdown, (n)	4
Post-test counseling	
By primary physician, n (%)	30 (37.5)
The time between diagnosis and counseling (median (IQR) days)	4 (1, 9)
Gestation age at post-test counseling (median (IQR) days)	19 (17, 22)

Delay in getting tests done was highly variable and affected by education status. Duration between advising and getting the ultrasound done was significantly longer among illiterate women (41 ± 22.6 days) than women educated up to 12th standard (2.33 ± 5.6 days; p = 0.001), and among women belonging to lower SES as compared to upper middle SES (12.5 ± 17.3 days vs. 1.69 ± 3.2 days; p = 0.03).

Delay in post-test counseling

**Figure 2:**
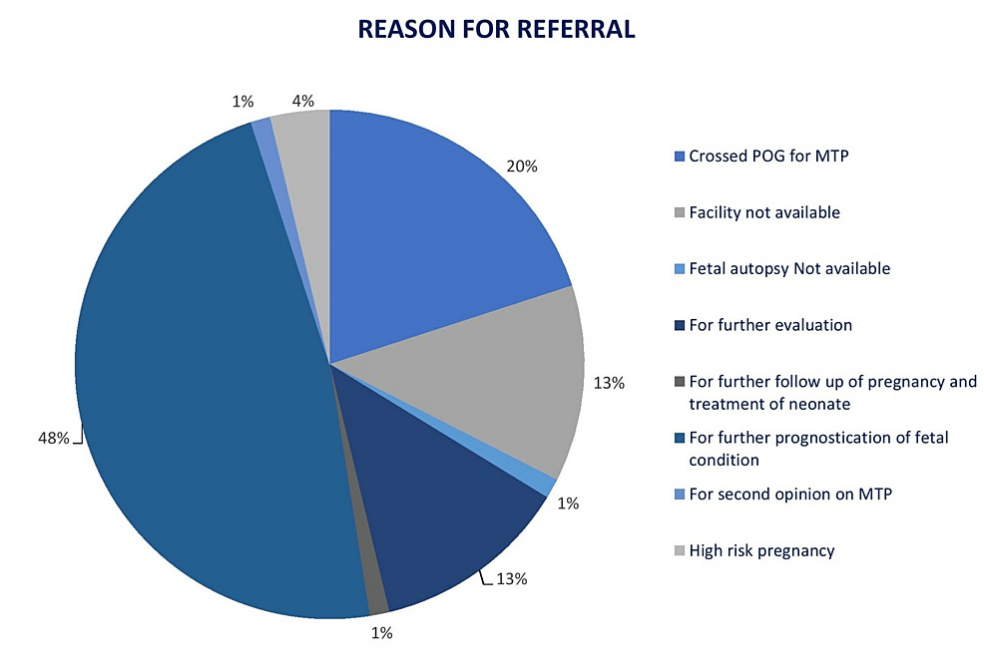
Reason for referral as cited by the referring physician POG: period of gestation; MTP: medical termination of pregnancy.

Most women (70%) returned to the primary physician within two days of diagnosis, while it took more than a week for eight women. Only 30 out of 80 (37.5%) women were counseled by their primary care provider about the fetal anomaly. The rest of them were referred on various grounds without any counseling. Figure 2 elaborates on the reasons for referral as cited by their physician, the most common being prognostication (47.50%).

Many women expected that they were being referred for treatment of fetal anomaly. "There is some problem in baby for which we have been sent to you… will it get treated?" was a common query. The timelapse between the prenatal diagnostic test and post-test counseling varied between zero and 40 days.

Barriers faced by women in seeking TOPFA

Owing to delay at different levels, 31 women (38.7%) could be counseled about fetal abnormality for the first time after 20 weeks, while another nine were counseled after 24 completed weeks. Since this study was carried out before the implementation of the MTP Amendment Act in March 2021, these women could not be offered abortions. Of these, 17 women were able to obtain permission for an abortion from a court of law.

**Figure 3:**
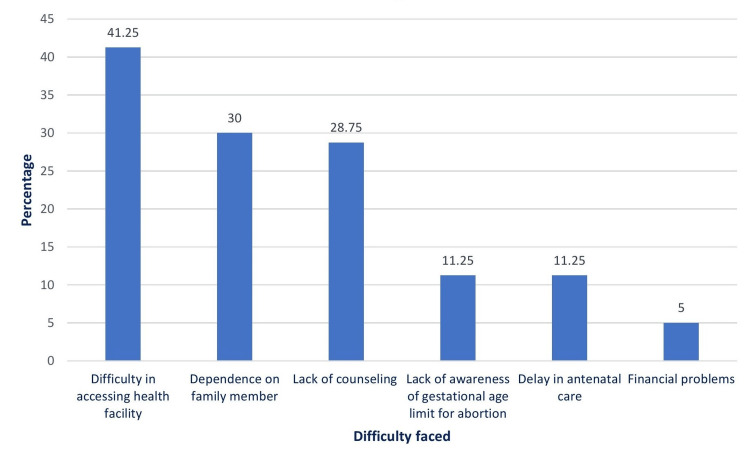
Barriers faced by women that led to delays in seeking abortion

Figure 3 shows the problems perceived by women that lead to delays in tests and seeking an abortion. Most of them encountered multiple issues. Most of them (n = 33) found it difficult to arrange travel and get accommodation for a few days. In 24 cases, the main problem was dependence on family members (mainly the husband) because the husband had been working at a different place. The COVID-19 lockdown contributed to problems in seven cases. Many women attributed their problems to a lack of counseling, a lack of awareness of the gestational age limit, and a delay in seeking antenatal care. Only four women reported no difficulty in seeking an abortion.

## Discussion

With more than 75% of women entrusting their antenatal care to state-run public healthcare facilities, these facilities remain an essential provider of obstetric health care in developing countries. Since these facilities are directly under the control of the government, the quality of antenatal care can be audited and improved. Despite its proven role in preventing neural tube and cardiac defects and WHO recommendations [10], less than 50% of women enrolled in our study received folic acid during pregnancy. Late entry into the antenatal care program is one of the reasons, as nearly 26% of women sought the first antenatal consultation after the first trimester. This delay in antenatal care also results in a delay in prenatal testing. Only 21 women (26%) had undergone aneuploidy screening, of whom three had abnormal results that were further confirmed by the diagnostic tests. A larger proportion of patients might have been diagnosed with a fetal anomaly at an earlier gestation had they undergone testing earlier. In one large study, 213 out of 488 fetal abnormalities (43.6%) were detected at 11-13 weeks, and 34% of fetuses with major cardiac anomalies had increased nuchal translucency [11]. According to the American College of Obstetricians and Gynecologists guidelines, aneuploidy screening should be offered to all pregnant women [12]. In our study, 27 women expressed that they would have preferred such tests over the agony of late diagnosis. On the other hand, 17 women opted out of the screening test despite being offered mainly due to financial reasons and lack of family support. Presently, routine screening and testing for aneuploidies and structural anomalies in the first trimester in low-risk pregnancies are difficult to sustain in resource-constrained settings. Financial concerns should be weighed against the high risk of complications and psychological stress associated with abortion at later gestation. Hence, until these tests are made freely available in the public sector, they should be offered to all women, who may or may not opt, depending on their affordability.

Most fetal structural abnormalities can be detected before 20 weeks of gestation [13]. None of the women in our study had such fetal malformations that could not have been detected by 18-19 weeks of gestational age, while many could have been diagnosed by the end of the first trimester. A study from a single medical center in Israel concluded that more than half of the late abortions for fetal abnormalities could have been done earlier [14]. The diagnosis was delayed beyond 20 weeks in 35 (43.75%) women in our study. Reasons for delay in prenatal tests were interlinked. Social and personal barriers prevented many women from reporting at appropriate gestation and getting the test done in time. Non-availability of diagnostic tests locally and dependence on family members put forward a combination of administrative and social barriers. Delay in the diagnostic test is perceived negatively by the couples and adds to their anxiety and frustration [3]. Pre-test counseling regarding the need for tests, approximate turnaround time, and implications of the abnormal result must be imparted. Decoupling counseling from testing was the reason for the delay in nearly 25% of cases in our study.

We found post-test counseling to be largely inadequate. Only 37% of all the women in our study were adequately counseled about fetal anomaly by their primary clinician such that they were aware of the diagnosis of anomaly and its prognosis. In most referral slips, only “Referred to Higher Center” was mentioned without providing a clear indication. Handover of incomplete information keeps the care providers at the referral center ill-informed and delays further action. Overall, 48% of women in our study were referred to ascertain the fetal prognosis, and another 13% of women were referred to our institute for repeat testing without being counseled about the existing results. Just informing that “something is not right with the baby” leads to confusion. Couples who are not well-informed are less well-prepared physically and psychologically and remain unsure of what to expect. Many women thought that they were being referred for treatment of fetal anomaly. More than 62% of women were counseled about abnormality for the first time at our center. Appropriate and timely counseling helps them make informed choices in time and allows them time to arrange funds/make preparations.

These preventable delays in diagnosis and post-test counseling pushed the decision for abortion beyond 20 weeks in 50% of cases. All these women were denied an abortion because the upper legal limit of gestation for abortion was 20 weeks before new amendments came into force in March 2021 [4]. This forced many of them to explore legal action whereby they pled to the court of law for permission for abortion, thus adding to their agony. Seventeen women could undergo termination after getting approval from a court of law, but the rest of them apparently continued pregnancy against their wishes. Restrictive abortion laws cause a woman to seek unsafe abortions [15], which account for 13% of maternal deaths globally. In countries like Belgium, Germany, and the Netherlands, where abortion is legal, the abortion rate is below 10 per 1,000 women. In Africa, Latin America, and the Caribbean, where abortion laws are restrictive, the rates are up to 39 per 1,000 women [16]. Legal restrictions do not reduce the number of abortions; they convert abortion into an unsafe one.

Though the upper gestational age limit for TOPFA has now been relaxed in India, this should not be interpreted as liberty to delay prenatal diagnosis. Second-trimester abortions are responsible for two-thirds of all abortion-related major complications [17], including post-abortion hemorrhage, cervical laceration, uterine perforation, and infection [18], and the complication rate increases with every passing week. A deficient provider base is also an essential barrier in developing countries. A survey in 221 districts across India reported that only 13% of primary health centers (PHCs) had a trained doctor to perform an abortion [19]. According to a large-scale study conducted in six Indian states, just 11-32% of all abortions occurring annually in respective states were performed in healthcare facilities. Only 27-48% of above-PHC-level public facilities (community health centers and hospitals) provide abortion services [20]. The provider base for abortion services shrinks further when it comes to second-trimester abortions [19-21]. Women with a gestational age of more than 12 weeks are referred away from PHCs [19]. Private facilities, too, are reluctant to provide second-trimester abortions. According to one study, 78% of private facilities refer away the women seeking second-trimester abortions, mainly to government hospitals [21]. Thus, delay in seeking abortion makes access even more difficult. Difficulty locating a provider or raising funds for abortion has been identified as the main obstacle in a study done at six facilities in Michigan and New Mexico [22]. Though “lack of facilities for abortion” was cited as the reason for referral in 13% of cases in our study, others were also counseled by the referring physicians not to return for further care or abortion.

Financial constraints, as a barrier to abortion, became further magnified by the lockdown due to COVID-19 because of restrictions on movement and loss of jobs. In a survey of 1,209 patients who underwent abortions at 11 large providers in the US, the main reason cited for delay in obtaining an abortion was time taken for making arrangements (59%), primarily financial. Underprivileged women were two times more likely to be tardy [23]. A study from Nairobi, Kenya showed that women sought abortion services from unqualified providers due to a lack of financial support, perceived illegality, and cultural beliefs [24].

The distress due to the diagnosis of fetal anomaly and a woman’s disinclined demand for abortion is further compounded by the denial of abortion. This leaves these women embittered due to the compulsion to continue the pregnancy and the arbitrary system they have to negotiate [25]. The problem in accessing abortion for fetal anomalies is multi-pronged and stems from the ineffective system of prenatal diagnosis, counseling, and access to abortion. Relaxation in the gestational age limit for abortion lifts one barrier and brings women closer to safe abortion by one step. This should not be dissociated from generating awareness, providing counseling, improving infrastructure, and capacity-building at public healthcare facilities for timely abortions because abortion-related complications and mental trauma are directly related to gestational age.

## Conclusions

Public healthcare facilities are the primary providers of antenatal care. Delay in diagnosis of a fetal anomaly is the major reason for the delay in the decision for abortion. This is primarily due to delays in seeking antenatal care, irregular follow-up, and lack of counseling. Once the fetal anomaly is diagnosed, most of the women are just referred to another center without counseling about the result and possible need for abortion. Primary healthcare providers should provide pre- and post-test counseling.

There is a need to strengthen the existing healthcare facilities, expand the provider base, and encourage capacity-building training because most women find it difficult to approach another facility for an abortion. Most women seeking TOPFA suffer agony and anxiety over delay in diagnosis, lack of awareness, and lack of counseling by their physician.
